# Electric field imaging of single atoms

**DOI:** 10.1038/ncomms15631

**Published:** 2017-05-30

**Authors:** Naoya Shibata, Takehito Seki, Gabriel Sánchez-Santolino, Scott D. Findlay, Yuji Kohno, Takao Matsumoto, Ryo Ishikawa, Yuichi Ikuhara

**Affiliations:** 1 Institute of Engineering Innovation, School of Engineering, The University of Tokyo, Yayoi 2-11-16, Bunkyo-ku, Tokyo 113-8656, Japan; 2 School of Physics and Astronomy, Monash University, Melbourne, Victoria 3800, Australia; 3 JEOL Ltd., 1-2-3 Musashino, Akishima, Tokyo 196-8558, Japan; 4 Nanostructures Research Laboratory, Japan Fine Ceramic Center, 2-4-1 Mutsuno, Atsuta-ku, Nagoya 456-8587, Japan

## Abstract

In scanning transmission electron microscopy (STEM), single atoms can be imaged by detecting electrons scattered through high angles using post-specimen, annular-type detectors. Recently, it has been shown that the atomic-scale electric field of both the positive atomic nuclei and the surrounding negative electrons within crystalline materials can be probed by atomic-resolution differential phase contrast STEM. Here we demonstrate the real-space imaging of the (projected) atomic electric field distribution inside single Au atoms, using sub-Å spatial resolution STEM combined with a high-speed segmented detector. We directly visualize that the electric field distribution (blurred by the sub-Å size electron probe) drastically changes within the single Au atom in a shape that relates to the spatial variation of total charge density within the atom. Atomic-resolution electric field mapping with single-atom sensitivity enables us to examine their detailed internal and boundary structures.

## Abstract (web summary)

The ability of scanning transmission electron microscopy (STEM) to image single atoms is becoming increasingly sophisticated. Here, the authors use differential phase contrast STEM to map the atomic electric fields within single Au atoms and SrTiO_3_ crystals, a step toward visualizing such intra- and interatomic electronic structure as chemical bonds.

Single-atom imaging by an electron microscope was first realized by Crewe *et al*.^1^ using scanning transmission electron microscopy (STEM), and STEM developments have pursued direct single-atom imaging with higher resolution and sensitivity ever since^2^,^3^. Through the advent of aberration correction technology^4^,^5^, annular dark field (ADF) STEM imaging has become a commonly used technique for imaging single atoms and thereby solving a wide range of scientific and technological problems^6^,^7^,^8^,^9^,^10^,^11^. In this imaging mode, the doughnut-shaped, post-specimen annular detector selectively collects high-angle scattered electrons at each probe position in a raster scan to form images, as schematically shown in Fig. 1a. Since electrons must pass close to the dense, positively charged atomic nucleus to scatter through large angles, atoms manifest as intensity peaks in STEM images of high-angle scattering. This image formation mechanism has made ADF imaging a powerful technique for determining precise atom positions with strong atomic-number-dependent contrast^12^. However, the information contained in these images is limited primarily to the positions of atomic nuclei; information about the surrounding electrons is minimal because the nuclear charge screening they provide has minimal effect on the high-angle electron scattering.

Differential phase contrast (DPC) imaging has long been used in STEM to directly visualize local electromagnetic fields inside materials^13^,^14^,^15^,^16^,^17^,^18^,^19^,^20^. Owing to the recent rapid progress in high-sensitivity area detectors^21^, it is becoming possible to use DPC STEM imaging at atomic resolution to probe the electric fields of single atomic columns in crystals^22^,^23^, as indicated schematically in Fig. 1a,b. When the fast, atomically sharp electron probe penetrates the atomic electron cloud, it is deflected by the net electric field between the atomic nucleus and the surrounding electron cloud. This deflection changes the electron flux intensity detected by each segment in the post-specimen segmented-type detector (Fig. 1b). This intensity variation—more precisely the variation of the centre of mass (CoM) of the electron distribution in the diffraction pattern^23^,^24^,^25^—at each raster position allows us to map out in real-space the atomic electric field of atomic columns and, potentially, of individual atoms. The atomic electric field, the field between the atomic nucleus and the surrounding electron cloud, should possess information about the atomic species, local chemical bonding and charge redistributions in between bonded atoms.

It has been proposed that atomic-resolution DPC STEM is sensitive to the ionicity of atoms in crystals^22^, and has potential advantages over other existing techniques in transmission electron microscopy (TEM) with sensitivity to atomic-scale charge redistribution effects, such as high-resolution TEM^26^, convergent beam electron diffraction^27^ and electron holography^28^. One advantage is the availability of simultaneous, complementary signals during DPC STEM imaging (for example, ADF and X-rays) that facilitate the characterization of local atomic structures and chemical information from exactly the same area. Another advantage is that segmented-detector DPC STEM can reconstruct an electric field vector map and a charge density map from single set of data in real-time without elaborated post-image processing. Yet another advantage is that DPC STEM does not require high-defocus conditions to obtain phase contrast, allowing structural features (ADF) and electromagnetic field structures to be observed simultaneously. However, the practical experiments are still extremely challenging, especially for single atoms, because of limitations in the sensitivity, stability and speed of data acquisition and processing using current instrumentation, preventing the application of this technique to many important scientific problems.

In the present study, we use a newly developed, high-speed, segmented detector to demonstrate real-space imaging of atomic electric fields, from columns of several atoms in a SrTiO_3_ single crystal to individual single Au atoms. By carefully controlling the imaging characteristics of the segmented detector, we show that atomic electric field mapping within isolated single atoms is possible to a very good approximation. This opens new possibilities for atomic-resolution electron microscopy, from merely seeing atom positions to visualizing detailed intra- and interatomic electronic structures of isolated and bonded atoms. This capability may lead to the direct imaging of local charge redistribution at a single-atom level, which might cast new light on our understanding of the properties of single atoms and small clusters on functionalized supports.

## Results

### Atomic-resolution DPC STEM of a SrTiO_3_ single crystal

**Figure 2:**
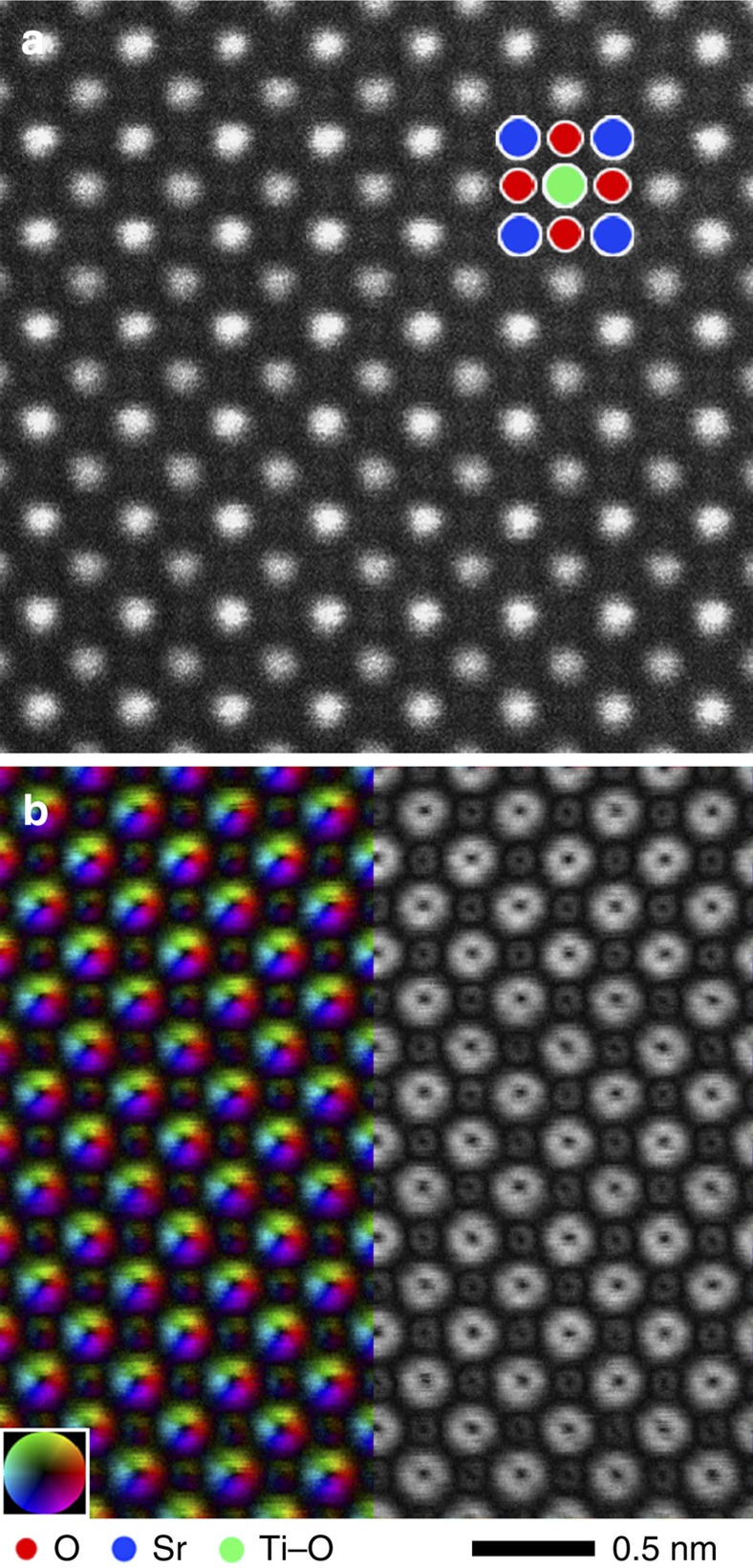
Simultaneous atomic-resolution STEM images of SrTiO_3_ [001]. (**a**) ADF STEM image. (**b**) Projected electric field vector colour map (left side) and electric field strength map (right side) constructed from the segmented-detector STEM images. The inset colour wheel indicates how colour and shade denote the electric field orientation and strength in the vector colour map. It is seen that both heavy and light element columns are sensitively imaged. Intensity dips are clearly visible at the centre of each atomic column position.

Figure 2 shows simultaneously acquired atomic-resolution ADF and DPC STEM images of a SrTiO_3_ single crystal observed from the [001] direction. These images are obtained with an aberration-corrected STEM (JEOL ARM-300CF, 300 kV) equipped with a newly developed, high-speed, segmented detector. The optical conditions and detector settings are described in the Methods section and Supplementary Fig. 1. These images constitute the average, after alignment, over very fast scan STEM images acquired in 10 sequential frames, each containing 1,024 × 1,024 pixels at a dwell time of 4 μs per pixel. This process significantly reduces image drift while improving signal-to-noise ratio^29^. Figure 2a shows the ADF STEM image. The strong and weak-intensity peaks correspond to the Sr and Ti–O atomic columns, respectively. Oxygen atomic columns are only faintly visible in the ADF image because of their weak scattering power at higher angles. Using eight detector segment images as shown in Supplementary Fig. 2, the CoM of the diffraction pattern on the detector is estimated for each raster position^24^ (the detailed processing is described in the Methods). The left hand side of Fig. 2b shows the constructed projected electric field vector map, or, more precisely, the local electric field map blurred by the intensity distribution of the sub-Å electron probe used in the experiment. The colour contrast corresponds to the relative direction and strength of the electric field at each raster position in the image. Comparing with the simultaneous ADF image, disks of rotating colour contrast are seen at each atomic column position, including the oxygen columns, in the electric field vector map. This reinforces the fact that the electric field vector map is sensitive to both heavy and light element atomic columns^23^,^24^. The direction of rotating colour contrast is the same in all the atomic columns irrespective of the atomic species, indicating that the (projected) atomic electric field points outwards from the centre of the atomic columns. The right hand side of Fig. 2b shows the electric field strength map constructed from the segmented-detector STEM images, the image contrast indicating the strength of the (projected) in-plane electric field at each raster position, that is, the modulus of the vector field shown on the left in that figure. The electric field strength map of each atomic column has a local intensity minimum at its centre because the projected in-plane component of the atomic electric field should be zero at the centre of the atomic columns, where the field is parallel to the incident electron beam direction. It should be noted that these electric field vector and strength maps can be constructed in real-time, simultaneously with the recording of the atomic-resolution segmented detector and ADF STEM images, as shown in the Supplementary Movie 1 (explained in Supplementary Fig. 3). The 512 × 512 scanning pixel movie was recorded with a dwell time of 3 μs per pixel, or less than 2 s per frame (including fly back time), and shows electric field vector maps of Sr, Ti–O and O columns clearly. Using a charge-coupled device detector would give much finer detail in the scattering distribution, but at the expense of significantly greater recording time: a recent report on electric field mapping in SrTiO_3_ (ref. 23) showed a 20 × 20 scanning pixel image with a dwell time of 50 ms per pixel, or 4 min for the single electric field vector map (including drift compensation, but excluding the data post-processing). Since STEM imaging is always susceptible to electronic noise, sample drift, damage and contamination during scanning, the ability to construct atomic-scale electric field maps during a rapid scan should be essential for characterizing local structures such as single atoms, clusters and interfaces and opens the door to real-time visualization of electromagnetic fields in *in situ* experiments. However, in both segmented detector and pixelated detector cases, dynamical electron scattering hampers a simple connection between CoM, and projected electric field such that detailed comparison with image simulations is necessary for full quantitative analysis in crystalline materials^22^,^23^,^24^.

**Figure 3:**
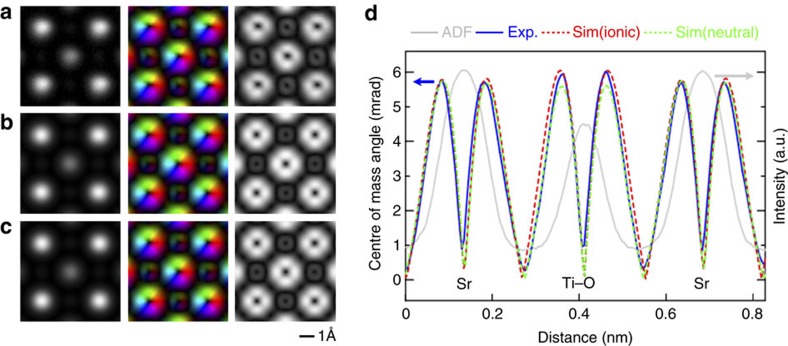
Quantitative comparison between experimental and simulated images. (**a**) Experimental repeat-unit averaged ADF (left), electric field vector (centre) and electric field strength (right) images. (**b**,**c**) Simulations for these same imaging modes based on ionic and neutral atom potentials, respectively. These simulations assume identical imaging conditions to the experiment, with a sample thickness of 8 nm and a defocus value of −5.1 nm (underfocus). (**d**) The normalized intensity profiles across the same Sr and Ti–O atomic columns in the averaged images from the ADF and electric field strength maps. While the ADF profile (grey) has been normalized for convenience, the experimental electric field strength (blue) and simulated electric field strength profiles, labelled Sim(ionic) and Sim(neutral) (red and green, respectively), are shown on the same absolute scale, that is, CoM angle units. It is seen that the Sim(ionic) profile shows better quantitative agreement with the experimental electric field strength profile than the Sim(neutral) profile. Note, too, that the intensity dips in the electric field strength are in identical positions to the ADF intensity peaks. Moreover, the full width half maxima of the intensity dips of the electric field strength are much narrower than those of the ADF intensity peaks.

To determine whether the observed atomic electric field contains information on the outer valence electron distribution, we performed dynamical image simulations based on the frozen phonon model^30^ to explore the sensitivity of atomic-resolution DPC STEM to charge redistribution and bonding. Figure 3a shows magnified, unit-cell-averaged ADF, electric field vector and electric field strength maps. Figure 3b,c shows the simulated images using ionic and neutral atom potentials, respectively. These theoretical simulations follow exactly the procedures used in the experiments (that is, the segmented-detector CoM approximation) to construct the electric field vector and strength maps. Thus, quantitative comparison between the experimental and simulated images is valid. In the simulation using ionic potentials, we assume that the structure consists of Sr^2+^, Ti^4+^ and O^2−^ ions, thereby including charge redistribution. By contrast, the simulations using neutral atom potentials assume that all the constituent atoms are neutral, meaning that there are no chemical bonds between them. Comparing Fig. 3a–c, there are almost no visible differences. In the experimental electric field strength map in Fig. 3a, faint diagonal line contrast crosses the centre of the atomic column positions. These are artefacts of our segmented-detector edges, the contrast transfer in these directions being weak because of the presence of detector edges along these directions. That even such faint contrast artefacts are reproduced in simulations reinforces the good contrast agreement between experiment and theory.

Further quantitative comparison between the experimental and simulated electric field strength is shown in the line profiles in Fig. 3d. The simulated profiles across the Sr and Ti–O columns based on both the ionic and neutral atom potentials are shown. It is apparent that, even using exactly the same optical conditions and sample thickness, the electric field strength profiles based on the ionic and neutral atom potentials differ slightly. In the present case, differences of almost 0.4 mrad in CoM angle are found at the peaks of electric field strength about the Ti–O columns. This suggests that charge redistribution can be detected experimentally, provided we use detectors sensitive enough to detect 0.4 mrad CoM differences. In Supplementary Note 1, we experimentally measured the relationship between the total electron-dose and the statistical errors in CoM angle detection for the segmented detector used in this study. From this relationship, the present electron-dose condition is estimated to be capable of detecting much finer CoM differences of 0.032 mrad. Our detector is thus more than sensitive enough to detect 0.4 mrad CoM differences. The experimental profile shows better agreement with simulations based on ionic potentials than those based on neutral potentials, consistent with the established nature of bonding in SrTiO_3_. More detailed comparisons with varying sample thickness are shown in Supplementary Note 2, but the general tendency is the same. Thus, we conclude that the atomic-resolution DPC STEM images are sensitive to charge redistribution.

Figure 3d also compares intensity profiles across the same Sr and Ti–O atomic columns in the electric field strength map and the ADF image. The intensity dips in the electric field strength map are in identical positions to the intensity peaks in the ADF image, indicating that the electric field strength map can equally be used to determine atomic column positions. Intriguingly, the full width half maxima of the intensity dips in the electric field strength map are much narrower than those of the intensity peaks in the ADF image, meaning that electric field strength maps should enable the determination of atomic column positions with very high precision. Although picometre column position determination has been achieved previously by fitting Gaussian functions to the broader ADF atomic columns, for example, by Yankovich *et al*.^31^, the sharper contrast profiles of the electric field strength map should facilitate such analysis.

### Atomic-resolution DPC STEM of isolated Au single atoms

**Figure 4:**
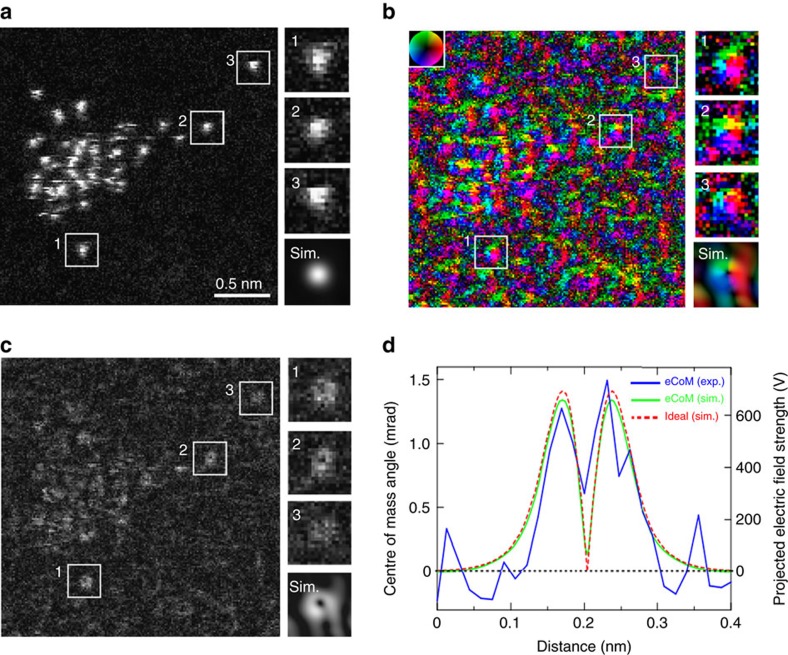
Simultaneous atomic-resolution ADF STEM image and electric field vector map and electric field strength map of Au single atoms. (**a**) ADF STEM image. (**b**,**c**) Electric field vector and electric field strength maps constructed from the segmented-detector STEM images. The dwell time is 300 μs per pixel. The inset colour wheel indicates how colour and shade denote the electric field orientation and strength. Magnified images from three isolated Au atom positions (3), identified from the ADF image but extractable from all the simultaneously acquired images, are shown to the right of the full images. The enlarged sections of the electric field vector and electric field strength maps show the distinctive contrast features seen at the column locations in Fig. 2. Simulated single Au atom images are also shown, which include a 10 nm-thick amorphous carbon substrate beneath the single Au atom. Because of the random structure of amorphous carbon, the diffraction effect is weak and thus the single Au atom contrast stands out from the background amorphous carbon contrast. (**d**) Comparison between the projected electric field strength line profile of Au atom number 2 and the simulated projected electric field strength line profiles of a single Au atom. For the experimental electric field strength line profile, the zero CoM angle is set to the average intensity of the nearby amorphous carbon region, and thus the comparison simulations do not include an amorphous carbon substrate. The experimental electric field strength using the eCoM approximation (blue line) and the simulated electric field profile assuming the same eCoM approximation (light green) are in good quantitative agreement. For comparison, the ideal atomic electric profile (including finite temperature effect) blurred by the diffraction-limited probe intensity profile and incoherent source size (red dashed line) is also shown. It is seen that the eCoM is a quantitatively good approximation to the (probe-blurred) atomic electric field.

The projected electric field strength is enhanced in crystalline materials viewed on axis because the atoms line up in columns. To demonstrate the ultimate sensitivity of quantitative DPC STEM electric field mapping down to the single-atom level, we use a model sample consisting of single atoms of Au dispersed on an amorphous carbon support film via a vacuum evaporation technique. The sample preparation is described in detail in the Methods section. The same method has been applied successfully to disperse noble metal single atoms on crystalline and amorphous substrates^32^,^33^,^34^. Figure 4a–c shows simultaneously imaged ADF, electric field vector and strength maps of Au single atoms, respectively. As detailed in the Methods section, a much lower electron-dose condition (∼3 pA) than that for the SrTiO_3_ case (∼27 pA) was used to minimize Au atom motion during the beam scan. Since Au atoms (*Z*=79) are much heavier than carbon atoms (*Z*=6), the ADF intensity of Au atoms stands out clearly as intensity peaks above the background contribution of carbon atoms in the supporting film. Indeed, many bright contrast peaks that correspond to Au single atoms and small clusters can be clearly seen in the ADF image. The distinction is less clear in the electric field vector map in Fig. 4b, since the range of colours complicates visual interpretation, and in the electric field strength map in Fig. 4c, since the electric field strength scales approximately as the atomic number, whereas the ADF image scales more strongly as the atomic number squared. However, using the ADF image as a reference for finding Au single-atom positions in the corresponding electric field vector and strength maps, we focus on three well-separated, isolated Au single atoms in the field of view. Numbered from 1 to 3, magnified ADF, electric field vector and strength maps of each atom are shown to the right of the full images. The electric field vector and strength maps at these atom positions show the distinctive contrast features identified in the SrTiO_3_ crystalline case. In particular, radially outward electric field contrast is again found in the electric field vector maps about the Au atom positions. However, there are many positions in Fig. 4b,c where the electric field vector and electric field strength maps show appreciable image contrast, but the ADF image does not. This contrast is considered to come from the amorphous carbon support. Since atomic-resolution DPC sensitively images the local electric fields, DPC variations arise not only because of the Au atoms, but also because of local sample thickness changes, surface steps and density variation of the amorphous carbon support. Therefore, single-atom imaging by DPC will be more susceptible to background contributions compared with ADF imaging. To consider the amorphous carbon support effect, we simulated electric field vector and strength maps of an Au single atom on a 10 nm-thick amorphous carbon substrate. The simulated images are shown below the magnified Au single-atom images. It is seen that, because of the supporting film's amorphous structure and the weak scattering power of its constituent carbon atoms, the Au single-atom contrast stands out from the background contrast. However, if single atoms were located in more strongly diffracting environments such as on crystalline substrates or within crystalline interfaces, diffraction effects would likely confound determination of the true electric field contributions. That diffraction can cause additional and misleading contributions to DPC image contrast at crystalline interface regions has been shown in the literature^35^. Nevertheless, in regions with minimal background contribution, such as on top of non-diffracting amorphous structures, this study shows that DPC imaging of single atoms is indeed possible.

Figure 4d compares the line profiles of the experimental (projected) electric field strength map for Au atom number 2 (blue line) and two theoretical electric field strength maps, one from effective segmented-detector CoM approximation simulations described below (light green line, labelled eCoM) and the other from direct calculation of the projected electric field of a neutral Au single atom (including finite temperature effect) convolved with the intensity distribution of the electron probe (red dashed line). Vertical axes are given in units of both CoM angle and projected electric field strength. For simplicity, these simulations ignore the effect of the amorphous carbon support. The effective segmented-detector CoM analysis is the modified version of the segmented-detector CoM approximation that involves fitting its phase-contrast transfer function to that of the pixelated detector CoM, thereby improving the accuracy of the CoM determination. A detailed derivation and discussion of the eCoM approximation is given in the Supplementary Note 3. It is seen that the experimental and theoretical electric field strength line profiles show quantitative agreement in both CoM angles and the estimated projected electric field strength. Going out from the atom centre, the theoretical electric field strength profile increases, turns around and decreases again, in a shape that relates to the spatial variation of total charge density within the Au single atom blurred by the sub-Å electron probe. Our experimental electric field strength exhibits a quantitatively similar profile, albeit perturbed by scan noise, indicating that the electric field vector and strength maps are indeed visualizing the atomic electric field of a single Au atom in real-space. To increase the accuracy of the electric field profile quantification beyond simple analysis using the present segmented detector, a pixelated detector^23^,^36^,^37^,^38^ could be used. However, pixelated detectors do not yet favour fast real-time imaging, and the difference between the simulated effective segmented-detector CoM approximation electric field profile and the ideal result, that is, the input-projected electric field blurred by the sub-Å electron probe, appears to be minimal in the case of single atoms as shown in Fig. 4d and Supplementary Fig. 10.

In summary, we have demonstrated real-space electric field mapping of SrTiO_3_ single crystal and single Au atoms by atomic-resolution DPC STEM. It was shown that atomic-resolution DPC STEM with high-speed segmented detectors can visualize atomic electric fields within atomic columns of crystals and even within isolated single atoms to a very good approximation. Atomic-resolution DPC STEM is shown to be sensitive to charge redistribution, probing the ionic bonding nature of SrTiO_3_. Atomic electric field imaging is shown to further be sensitive to both heavy and light element atoms within a crystalline environment, and to allow for the determination of atomic column positions with very high precision. The new ability shown here opens an alternative way for directly visualizing atoms and nanostructures, that is, seeing atoms as an entity of electromagnetic fields that reflect the intra- and interatomic electronic structures.

## Methods

### Sample preparation

Commercially available stoichiometric SrTiO_3_ single-crystal substrates were used as a starting material (Furuuchi Chemical Corporation, Japan). The substrates were cut and mechanically polished with diamond suspension to have a total thickness of less than 50 μm. The samples were then dimpled using a dimple grinder to obtain a thin area at the centre of the sample (less than 10 μm). A standard Ar ion-beam thinning method was used to obtain electron transparency. To minimize surface damage and contamination, the accelerating voltage was gradually decreased from 5 kV to less than 1 kV as thinning progressed. For the Au single-atom sample, high-purity gold (99.95%) was vacuum-evaporated onto a carbon-coated copper grid at a base pressure of ∼1 × 10^−7^ Pa at room temperature. It has been confirmed that this method has been successfully applied to the evaporation of Pt and Au single atoms on crystalline (TiO_2_) and amorphous carbon substrates^32^,^33^,^34^. The evaporation rate and time used here were 0.04 atom s^−1^ nm^−2^ and 10 s, respectively. The total evaporation amount of Au is far below the coverage of an Au monoatomic layer on the substrate surface.

### Atomic-resolution DPC STEM imaging

Atomic-resolution ADF and DPC STEM images were obtained simultaneously using a 300 kV aberration-corrected STEM (JEOL ARM-300CF) equipped with a second generation segmented annular all field detector with 16 segments. A detailed description of the segmented annular all field detector has been reported previously^21^. Supplementary Fig. 1 shows the relative orientation relationship between the SrTiO_3_ crystal and the detector segments. The probe-forming aperture semiangle was set to be 24 mrad. The spot size used was 8 c. The probe current used is estimated to be ∼27 pA by the Faraday cup. The angular ranges from the optical axis of detector segments 1–4 and 5–8 were 0–16 and 16–32 mrad, respectively, while the azimuthal span was 90 degrees. The same optical setting was used for the Au single-atom imaging, but the image and the detector orientation was rotated by 45 degrees from that used for the SrTiO_3_ analysis, as shown in Supplementary Fig. 4. The spot size used was 10 c. To minimize Au single-atom motion due to the probe scanning, we reduced the probe current significantly—to ∼3 pA as estimated by Faraday cup—compared with that used in the SrTiO_3_ case. Supplementary Figs 2 and 4 show the original simultaneous eight-segment images used for the electric field mapping of SrTiO_3_ and Au single atoms, respectively. Even in the original segment images, Au single atoms can be recognized, as indicated by white rectangles.

On the basis of the segmented-detector CoM approximation described in detail in ref. 24, eight segment images were used to estimate the CoM for the electric field vector and electric field strength mapping. The estimation of the CoM (*I*_CoM_) in the *x* direction (the *y* direction form follows trivially) was using the following equation^24^,


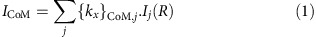


where {*k*_*x*_}_CoM,*j*_ is the *x* coordinate of the CoM of detector segment *j* and *I*_*j*_*(R)* is the electron intensity detected by the detector segment *j*. On the scale set by the extent of the bright-field disk in the detector plane, which depends on the probe-forming aperture size and the camera length, the {*k*_*x*_}_CoM*,j*_ can be determined geometrically. By calculating the CoM in the *x* and *y* directions using experimentally obtained segment images, we estimate the two-dimensional (2D) CoM on the detector plane at each raster position. Moreover, fitting the 2D phase-contrast transfer function of the present segmented-detector CoM approximation to that of pixelated detector CoM (which, for the conditions used, amounts to just slightly increasing the {*k*_*x,y*_}_CoM*,j*_ values) improves the accuracy of the CoM determination, as shown in Supplementary Figs 9 and 10. This approach, which we refer to as the eCoM approximation, is discussed in more detail in Supplementary Note 3.

### Image simulation

The image simulations were performed using the multislice method based on the frozen phonon model. The microscope parameters used are consistent with the experimental values. A 300 keV, aberration-free (excepting defocus) probe with probe-forming aperture semiangle of 24 mrad was assumed.

SrTiO_3_ image simulations were performed based on the scattering factors for both ions^39^ and neutral atoms^40^. The detector angles and orientation relative to the SrTiO_3_ crystal were as per the experiment. The thickness value was experimentally determined to be 8±1 nm by using the position-averaged convergent beam electron diffraction (PACBED) pattern obtained exactly from the observed area as described in Supplementary Note 4. The defocus value was determined via systematic matching with the experimental data to be −5.1 nm (underfocus). These parameters were used for the image simulations. Finite source size and probe instability were incorporated in the simulations via convolution with a Gaussian distribution, the half width half maximum of which was estimated to be 0.29 Å by fitting the simultaneously obtained ADF image intensity profile, as described in Supplementary Note 5.

For simulating the Au single-atom case, the potentials for neutral atoms^40^ were used, and the mean squared displacement <*u*^2^> was assumed to be 0.006 Å^2^. A defocus value of 0 nm was assumed since the experimental focus was set to obtain the best contrast in the simultaneously acquired ADF image. Finite source size and probe instability were incorporated in the simulations via convolution with a Gaussian distribution. The half width half maximum used was 0.25 Å, determined from the simultaneously acquired ADF image of Au atom number 2. The fitting result is shown in Supplementary Fig. 13. For the image simulation shown in Fig. 4a–c, we assumed a 10 nm-thick amorphous carbon structure under the Au single atom. For obtaining the ideal theoretical electric field profile (including finite temperature effect) shown as the dashed red line in Fig. 4d, the calculated electric field profile is blurred by (that is, convolved with) the diffraction-limited probe intensity profile and the Gaussian effective source distribution.

### Data availability

The data that support the findings of this study are available from the corresponding author upon request.

## Additional information

**How to cite this article:** Shibata, N. *et al*. Electric field imaging of single atoms. *Nat. Commun.* **8,** 15631 doi: 10.1038/ncomms15631 (2017).

**Publisher's note:** Springer Nature remains neutral with regard to jurisdictional claims in published maps and institutional affiliations.

## Supplementary Material

Supplementary Information Supplementary Figures, Supplementary Notes and Supplementary References.

Supplementary Movie 1 Live observation of SrTiO_3_ [001] by atomic-resolution DPC STEM.

Peer Review File

## Figures and Tables

**Figure 1:**
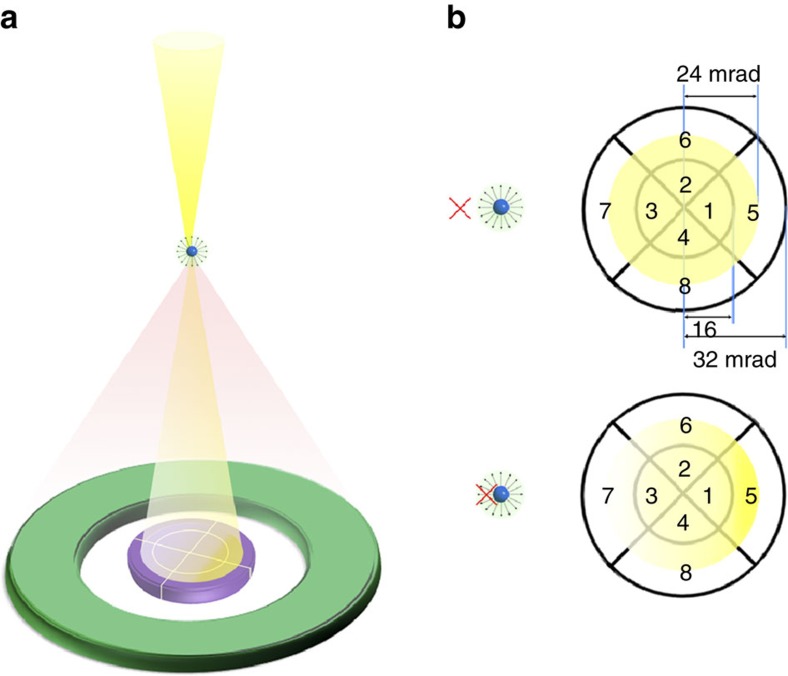
Schematic illustration of detector geometries in atomic-resolution STEM. (**a**) The large-angle doughnut-shaped detector (green) selectively collects electrons scattered to high angles by atoms to form atomic-resolution ADF STEM images. The segmented detector (purple), inserted in the bright-field region of the illuminating cone, sensitively detects atomic electric fields. (**b**) Schematic illustration of the bright-field disk intensity distribution on the segmented detector for two different electron probe positions, as indicated by red crosses, near an atom. When the probe position is away from the atom (upper figure), the bright-field disk intensity is uniform. When the probe position is in the vicinity of the atomic centre (lower figure), the intensity distribution changes because of the interaction of the electron probe with the perpendicular component of the atomic electric field (pointing radially from the atomic nucleus) as projected along the incident electron beam direction. The intensity change on the different detector segments can be used to determine the in-plane atomic electric field of an atom or atomic column.

## References

[b1] Crewe A. V., Wall J. & Langmore J. Visibility of single atoms. Science 168, 1338–1340 (1970). 17731040 10.1126/science.168.3937.1338

[b2] Pennycook S. J., Nellist P. D. (eds) in Scanning Transmission Electron Microscopy Springer (2011).

[b3] Tanaka N. (ed.) in Scanning Transmission Electron Microscopy of Nanomaterials Imperial College Press (2015).

[b4] Haider M. . Electron microscopy image enhanced. Nature 392, 768–769 (1998).

[b5] Batson P. E., Dellby N. & Krivanek O. L. Sub-angstrom resolution using aberration corrected electron optics. Nature 418, 617–620 (2002). 12167855 10.1038/nature00972

[b6] Wang S. . Dopants adsorbed as single atoms prevent degradation of catalysts. Nat. Mater. 3, 143–146 (2004). 14991014 10.1038/nmat1077

[b7] Allen J. E. . High-resolution detection of Au catalyst atoms in Si nanowires. Nat. Nanotech. 3, 168–173 (2008). 10.1038/nnano.2008.5 18654490

[b8] Oltalan V., Uzun A., Gates B. ,C. & Browning N. D. Towards full-structure determination of bimetallic nanoparticles with an aberration-corrected electron microscope. Nat. Nanotech. 5, 843–847 (2010). 10.1038/nnano.2010.234 21102466

[b9] Krivanek O. L. . Atom-by-atom structural and chemical analysis by annular dark-field electron microscopy. Nature 464, 571–574 (2010). 20336141 10.1038/nature08879

[b10] Hwang J., Zhang J. Y., D'Alfonso A. J., Allen L. J. & Stemmer S. Three-dimensional imaging of individual dopant atoms in SrTiO_3_. Phys. Rev. Lett. 111, 266101 (2013). 24483805 10.1103/PhysRevLett.111.266101

[b11] Ishikawa R. . Direct observation of dopant atom diffusion in a bulk semiconductor crystal enhanced by a large size mismatch. Phys. Rev. Lett. 113, 155501 (2014). 25375721 10.1103/PhysRevLett.113.155501

[b12] Pennycook S. J. & Boatner L. A. Chemically sensitive structure-imaging with a scanning transmission electron microscope. Nature 336, 565–567 (1988).

[b13] Rose H. Phase contrast in scanning transmission electron microscopy. Optik 39, 416–436 (1974).

[b14] Dekkers N. H. & de Lang H. Differential phase contrast in a STEM. Optik 41, 452–456 (1974).

[b15] Rose H. Nonstandard imaging methods in electron microscopy. Ultramicroscopy 2, 251–267 (1977). 888244 10.1016/s0304-3991(76)91538-2

[b16] Chapman J. N. The investigation of magnetic domain structures in thin foils by electron microscopy. J. Phys. D Appl. Phys. 17, 623–647 (1984).

[b17] Chapman J. N., McFadyen I. R. & McVitie S. Modified differential phase contrast Lorentz microscopy for improved imaging of magnetic structures. IEEE Trans. Mag. 26, 1506–1511 (1990).

[b18] Lohr M. . Differential phase contrast 2.0 – opening new ‘fields' for an established technique. Ultramicroscopy 117, 7–14 (2012). 22634135 10.1016/j.ultramic.2012.03.020

[b19] Shibata N. . Imaging of built-in electric field at a p-n junction by scanning transmission electron microscopy. Sci. Rep. 5, 10040 (2015). 26067359 10.1038/srep10040 PMC4464396

[b20] Matsumoto T. . Direct observation of Σ7 grain boundary core structure in magnetic Skyrmion lattice. Sci. Adv. 2, e1501280 (2016). 26933690 10.1126/sciadv.1501280 PMC4758740

[b21] Shibata N. . New area detector for atomic-resolution scanning transmission electron microscopy. J. Electron Microscopy 59, 473–479 (2010). 10.1093/jmicro/dfq014 20406732

[b22] Shibata N. . Differential phase-contrast microscopy at atomic resolution. Nat. Phys. 8, 611–615 (2012).

[b23] Müller K. . Atomic electric fields revealed by a quantum mechanical approach to electron picodiffraction. Nat. Commun. 5, 5653 (2014). 25501385 10.1038/ncomms6653 PMC4275586

[b24] Close R., Chen Z., Shibata N. & Findlay S. D. Towards quantitative, atomic-resolution reconstruction of the electrostatic potential via differential phase contrast using electrons. Ultramicroscopy 159, 124–137 (2015). 26381331 10.1016/j.ultramic.2015.09.002

[b25] Lubk A. & Zweck J. Differential phase contrast: an integral perspective. Phys. Rev. A 91, 023805 (2015).

[b26] Meyer J. C. . Experimental analysis of charge redistribution due to chemical bonding by high-resolution transmission electron microscopy. Nat. Mater. 10, 209–215 (2011). 21240288 10.1038/nmat2941

[b27] Zuo J. M., Kim M., O'Keeffe M. & Spence J. C. H. Direct observation of d-orbital holes and Cu-Cu bonding in Cu_2_O. Nature 401, 49–52 (1999).

[b28] Linck M., Freitag B., Kujawa S., Lehmann M. & Niermann T. State of the art in atomic resolution off-axis electron holography. Ultramicroscopy 116, 13–23 (2012).

[b29] Ishikawa R., Lupini A. R., Findlay S. D. & Pennycook S. J. Quantitative annular dark field electron microscopy using single electron signals. Microsco. Microanal. 20, 99–110 (2014). 10.1017/S1431927613013664 24168987

[b30] Kirkland E. J. Advanced Computing in Electron Microscopy Springer New York (2010).

[b31] Yankovich A. B. . Picometre-precision analysis of scanning transmission electron microscopy images of platinum nanocatalysts. Nat. Commun. 5, 4155 (2014). 24916914 10.1038/ncomms5155

[b32] Chang T.-Y., Ikuhara Y. & Shibata N. Effects of TiO_2_ Support on the initial stage of Pt nanoparticle growth. Appl. Phys. Exp. 6, 025503 (2013).

[b33] Chang T.-Y. . Direct imaging of Pt single atoms adsorbed on TiO_2_ (110) surfaces. Nano Lett. 14, 134–138 (2014). 24351061 10.1021/nl403520c

[b34] Matsunaga K. . Adsorption sites of single noble metal atoms on the rutile TiO_2_ (110) surface influenced by different surface oxygen vacancies. J. Phys. Condens. Matter 28, 175002 (2016). 27033403 10.1088/0953-8984/28/17/175002

[b35] MacLaren I. . On the origin of differential phase contrast at a locally charged and globally charge-compensated domain boundary in a polar-ordered material. Ultramicroscopy 154, 57–63 (2015). 25837677 10.1016/j.ultramic.2015.03.016

[b36] Waddell E. & Chapman J. Linear imaging of strong phase objects using asymmetrical detectors in STEM. Optik 54, 83–96 (1979).

[b37] Pennycook T. J. . Efficient phase contrast imaging in STEM using a pixelated detector. Part 1: experimental demonstration at atomic resolution. Ultramicroscopy 151, 160–167 (2015). 25458189 10.1016/j.ultramic.2014.09.013

[b38] Tate M. W. . High dynamic range pixel array detector for scanning transmission electron microscopy. Microsc. Microanal. 22, 237–249 (2016). 26750260 10.1017/S1431927615015664

[b39] Peng L.-M. Electron scattering factors of ions and their parameterization. Acta Cryst. A54, 481–485 (1998).

[b40] Peng L.-M., Ren G., Dudarev S. L. & Whelan M. Robust parameterization of elastic and absorptive electron atomic scattering factors. Acta Cryst. A52, 257–276 (1996).

