# Supplementary material for: Electric field imaging of single atoms
Source: Nat Commun. 2017 May 30;8:15631. doi: 10.1038/ncomms15631 (PMC5459997; doi:10.1038/ncomms15631)
Supplement: Supplementary Information — Supplementary Figures, Supplementary Notes and Supplementary References. [file ncomms15631-s1.pdf]

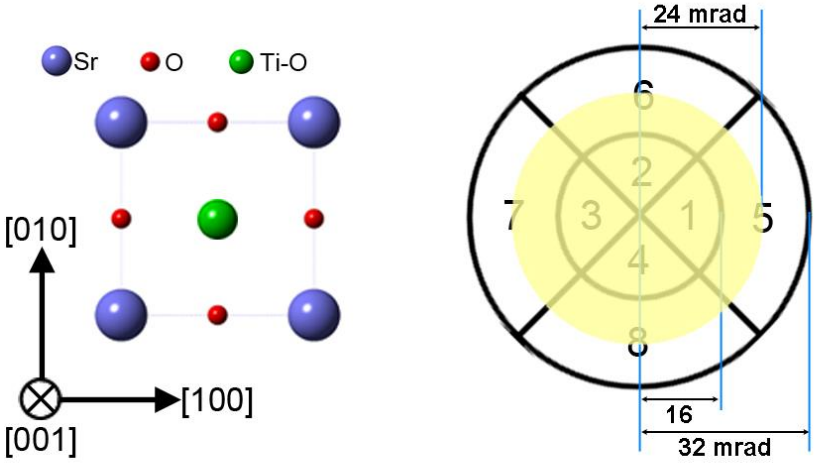

Supplementary Figure 1: **The relative orientation between $SrTiO_3$ crystal and segmented detector.** On the detector illustration, the bright-field disk is schematically shown as the yellow circle.

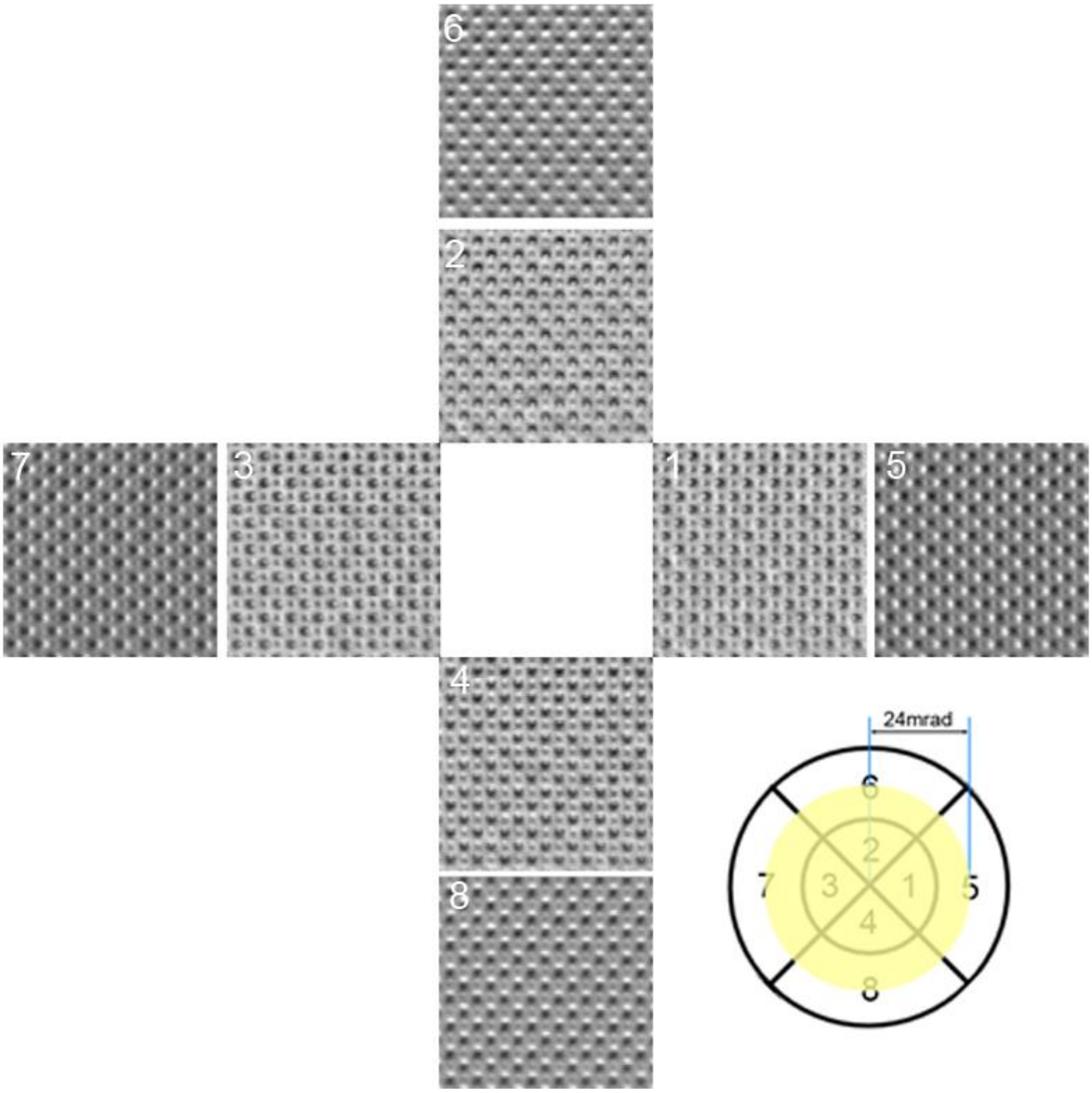

Supplementary Figure 2: **Eight simultaneously-acquired detector segment images used for the electric field mapping for $SrTiO_3$ in Fig. 2 and 3.**

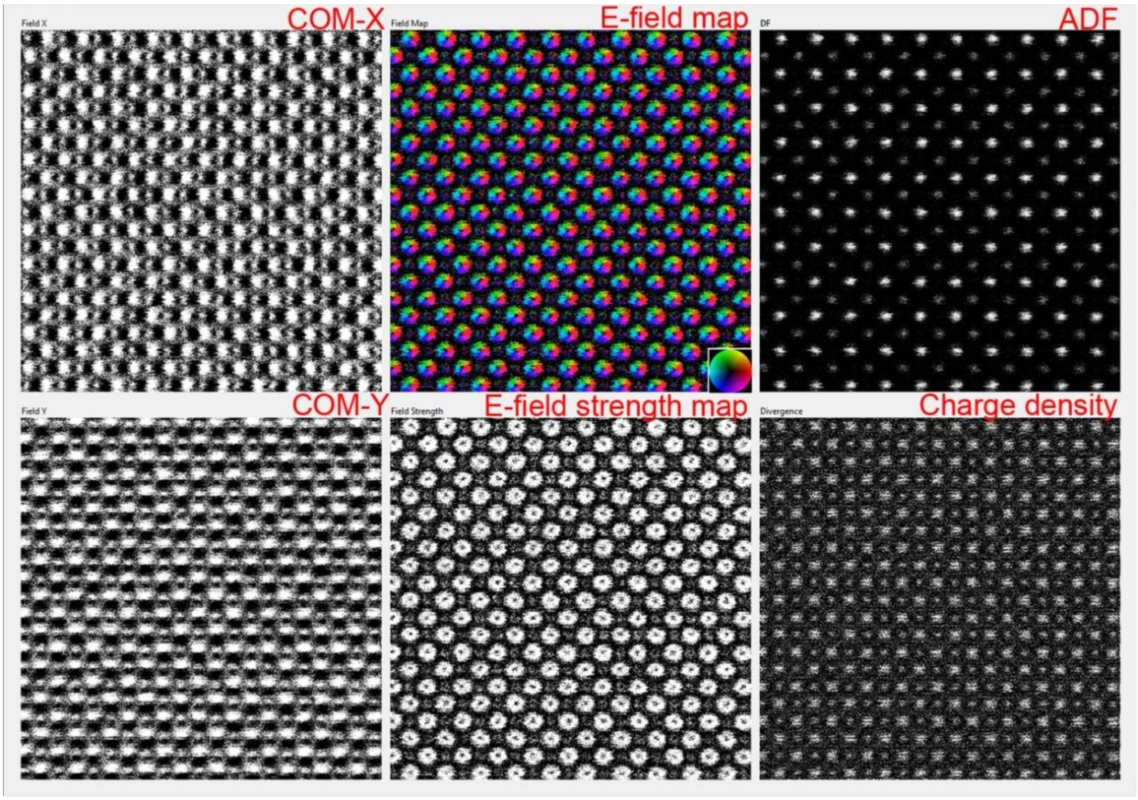

Supplementary Figure 3: **Description of the supplementary movie.** The supplemental movie shows the live observation of $SrTiO_3$ [001] by atomic-resolution DPC STEM. The dwell time is 3 μs/pixel. The two panels in the left column show the two DPC (center of mass) STEM images proportional to the in-plane electric field components in the two perpendicular directions based on the segmented detector center of mass approximation[1]. The two panels in the center column show the electric field color map (with inset color wheel indicating the electric field strength and direction) and the electric field strength map calculated from the two DPC STEM images. In the right column, the upper panel shows the ADF image and the lower panel shows the divergence of the electric field (charge density) map. In this case, the detector orientation with respect to the $SrTiO_3$ crystal orientation is 45 degree rotated from the images shown in Fig. 2 and 3.

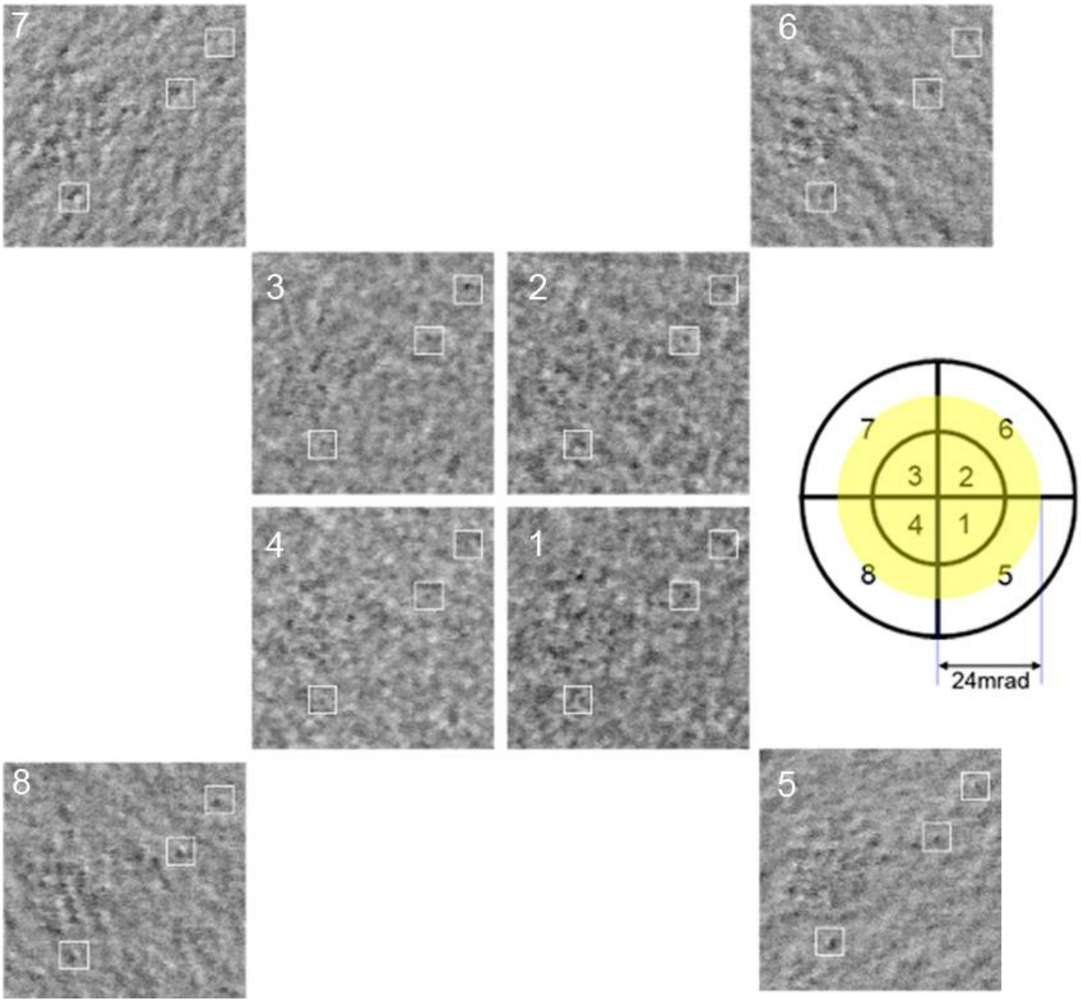

Supplementary Figure 4: **Eight simultaneously-acquired detector segment images used for the electric field mapping for single Au atoms in Fig. 4.**

Supplementary Note 1: Detector characterization

To estimate the sensitivity of the present DPC imaging to the CoM angles, we experimentally analyzed the SAAF detector used in this study. Supplementary Figure 5 shows the relationship between the standard deviation of the measured CoM angles and the electron dose. Here, we used the same optical settings as those for the $SrTiO_3$ and Au single atom imaging shown in the main text, but further independently measured this relationship with different spot size settings (and thus with different probe currents). It is seen that these three independent measurements lie on a straight line on this logarithmic plot: the standard deviation of the measured CoM angles and the electron dose show a simple inversely proportional relationship.

Using this experimental relationship, we can estimate the sensitivity of DPC STEM imaging to CoM angles under given electron dose condition.

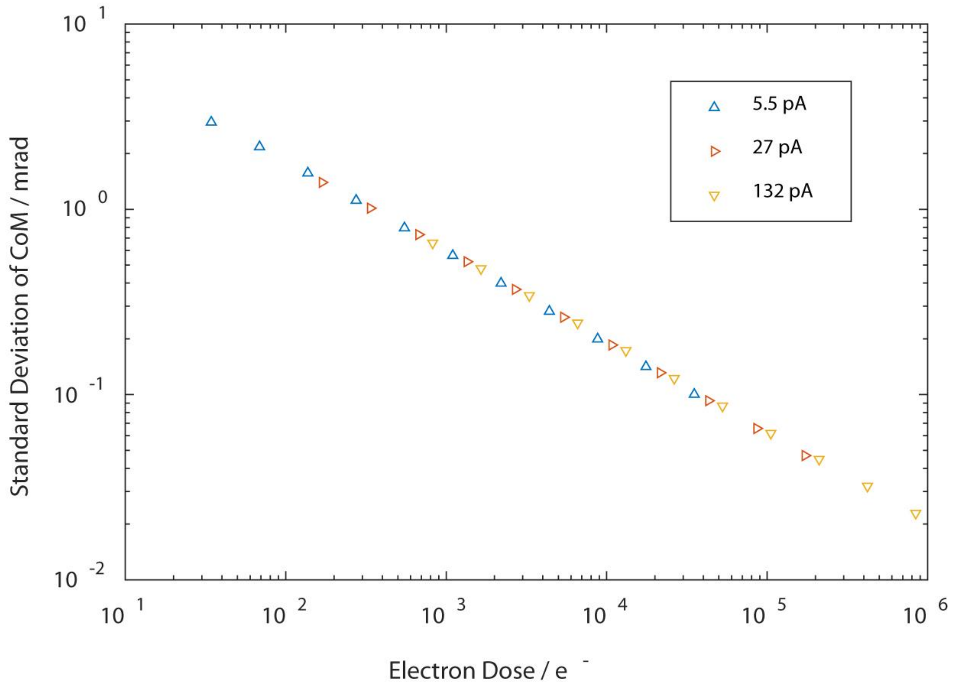

Supplementary Figure 5: The detector performance showing the relationship between the standard deviation of measured CoM angles and the electron dose.

Supplementary Note 2: Comparison between experiment and ionic / neutral potential image simulations with other thickness cases in $SrTiO_3$

Since the sample thickness estimation using PACBED yielded 8±1 nm as shown in Supplementary Note 4, we used 8 nm for image simulation in Fig. 3d. However, we have also performed the same comparison between experiment and simulation assuming 7 nm and 9 nm sample thickness cases, which are shown in Supplementary Figure 6 and Supplementary Figure 7, respectively. In each case, the defocus value used is that giving the best fit to the experimental profile. It is seen that the overall tendency is quite similar to the case of 8 nm. The profiles using ionic potentials clearly show better agreement with the experimental profile than those using neutral potentials. In particular, in the same imaging conditions, the profiles using neutral potentials tend to underestimate the electric field strength more than those using ionic potentials, especially in the vicinity of the Ti-O column. Although it is difficult to conclude which sample thickness is the best match, we can conclude that the profiles using ionic potentials well explain the experimental profiles in all three cases. Thus, in Fig. 3d, we plotted the simulated profile of 8nm using ionic potentials and neutral potentials as a representative.

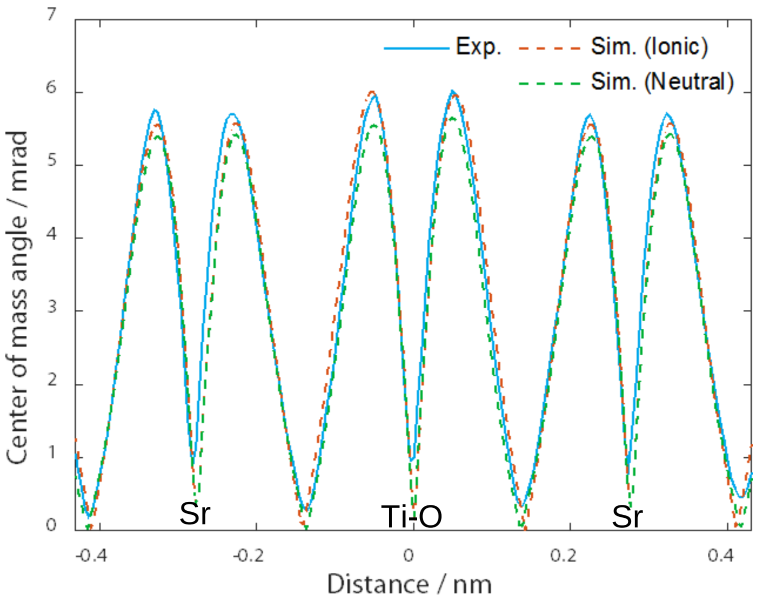

Supplementary Figure 6: Experimental and simulated electric field strength profiles of $SrTiO_3$. Here, we assume 7 nm sample thickness and defocus of -3.5 nm (underfocus).

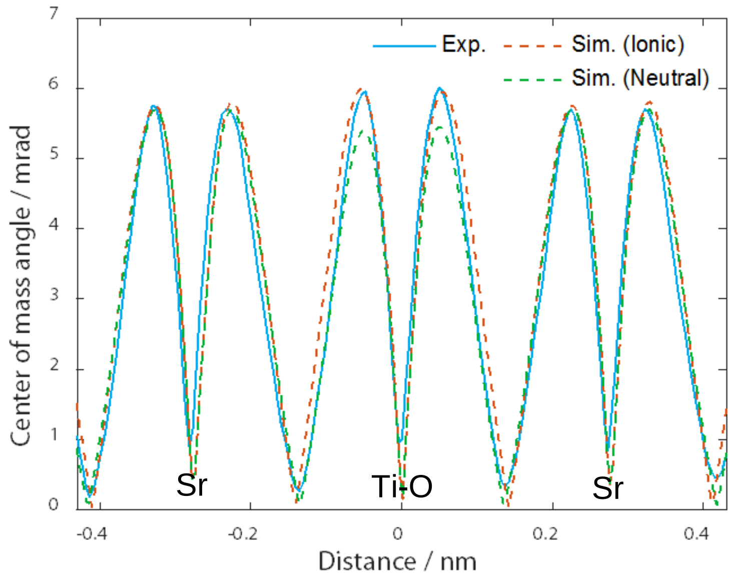

Supplementary Figure 7: Experimental and simulated electric field strength profiles of $SrTiO_3$. Here, we assume 9 nm sample thickness and defocus of -5.9 nm (underfocus).

Supplementary Note 3: Phase contrast transfer function analysis of the segmented-detector CoM approximation

Rose[2] has shown that, in the linear (or weak object) approximation, the phase contrast transfer function (PCTF) of general STEM imaging may be represented as follows,

$$L(\boldsymbol{\omega}) = \frac{i}{\Omega_0}\int A(\mathbf{K}_\perp)D(\mathbf{K}_\perp)\left(A(\boldsymbol{\omega}-\mathbf{K}_\perp)\exp\left[-i\left(\chi(\boldsymbol{\omega}-\mathbf{K}_\perp)-\chi(\mathbf{K}_\perp)\right)\right]\right.$$
$$\left. - A(\boldsymbol{\omega}+\mathbf{K}_\perp)\exp\left[i\left(\chi(\boldsymbol{\omega}+\mathbf{K}_\perp)-\chi(\mathbf{K}_\perp)\right)\right]\right)d^2\mathbf{K}_\perp \quad \cdots(1)$$

where $\boldsymbol{\omega}$ is a scattering wave vector and $\mathbf{K}_\perp$ is the component of incident wave vector perpendicular to optical axis in the equivalent TEM optical system according to the reciprocity theorem. The aperture function $A(\mathbf{K}_\perp)$ returns one if $\mathbf{K}_\perp$ lies within the condenser aperture, and zero otherwise. For usual STEM imaging with annular detectors, the detector function $D(\mathbf{K}_\perp)$ returns one if $\mathbf{K}_\perp$ lies within the detector and zero otherwise. However, more elaborate detector functions can be synthesised from segmented and pixelated detectors data by taking (possibly weighted) linear combinations of STEM images from different detectors / detector pixels. $\chi(\mathbf{K}_\perp)$ is the aberration function. Here, the normalizing constant Ω_0 is given by

$$\Omega_0 = \int A(\mathbf{K}_\perp)\, d^2\mathbf{K}_\perp \quad \cdots(2)$$

In the case of the segment-detector CoM approximation[1], we define the detector function for the x- component of the CoM as

$$D(\mathbf{K}_\perp) = \begin{cases} \{k_x\}_{\mathrm{CoM},j} & \text{if } \mathbf{K}_\perp \text{ lies within } j\text{th segment,} \\ 0 & \text{otherwise} \end{cases} \quad \cdots(3)$$

where $\{k_x\}_{\mathrm{CoM},j}$ is the x-coordinate of the center of mass of detector segment j.

In the case of the first-moment CoM, such as may be formed using pixelated detector data, we define the detector function for the x- component of the CoM as

$$D(\mathbf{K}_\perp) = \begin{cases} k_x & \text{if } \mathbf{K}_\perp \text{ lies within the detector,} \\ 0 & \text{otherwise} \end{cases} \quad \cdots(4)$$

where k_x is the x-coordinate of $\mathbf{K}_\perp$.

In the both cases, the PCTF $L(\boldsymbol{\omega})$ is a purely imaginary quantity. More conveniently, the PCTF $L'(\boldsymbol{\omega})$ can be defined as follows:

$$L(\boldsymbol{\omega}) = i\omega_x L'(\boldsymbol{\omega}) \quad \cdots(5)$$

where ω_x is the x-coordinate of $\boldsymbol{\omega}$. $L'(\boldsymbol{\omega})$ is the PCTF for the partial derivative of phase functions with respect to x.

Based on the above formulas, we calculated the PCTFs of the ideal pixelated detector and the segmented detector CoM in two-dimensions (since the PCTFs are not isotropic in two-dimensions). Supplementary Figure 8 shows the comparison between the PCTF along ω_x (for ω_y = 0) of the segment-detector CoM (green) and the pixelated-detector CoM (blue). This corresponds to a line profile across of the two-dimensional PCTF. It is seen that the PCTF of the segment-detector CoM has some deviation from that of the pixelated-detector CoM. To reduce this deviation, we fit the two-dimensional PCTF of the original segment-detector CoM approximation to that of the pixelated-detector CoM by scaling the $\{k_x\}_{\mathrm{CoM},j}$ using least-square fitting, assuming that the relative ratio of each $\{k_x\}_{\mathrm{CoM},j}$ is unchanged. This produces a modified PCTF for what we call the effective segment-detector CoM (eCOM) approximation, shown as the red line in Supplementary Figure 8. Although the good fit may not be so obvious in Supplementary Figure 8's line profile across the two-dimensional PCTF, the PCTF of eCOM indeed shows better agreement with the pixelated detector CoM overall. Using two different approximations – segmented-detector CoM and eCoM – we performed multislice frozen phonon simulations of electric field strength profiles within an Au single atom, and compare the results with the theoretical CoM (direct calculation of the time-averaged projected electric field of a thermally vibrating neutral Au single atom convolved with the intensity distribution of the electron probe). The results are shown in Supplementary Figure 9. The electric field profiles are basically similar between the segment-detector

CoM and the theoretical CoM, but the former appreciably underestimates the peak values. However, the electric field profile of the eCoM method shows much better agreement with the theoretical CoM. Quantification using the eCoM method is thus better than that using the original segment-detector CoM method. Note that, being based on the PCTF, the appropriate scaling may be determined for any given probe/detector configuration, independent of the sample. To support this idea, we performed systematic image simulation of electric field strength profiles for several different single atoms (C, Si, Cu, Ag) using the different detector approximations. The results are shown in Supplementary Figure 10. From light element to heavy element atoms, the segmented-detector CoM approximation always underestimates the peak CoM values. In contrast, the eCoM approximation always shows better agreement with the theoretical CoM irrespective of atom species. Thus, by adjusting $\{k_x\}_{\mathrm{CoM},j}$ to control the PCTF, we can improve the quantification of the segmented detector CoM approximation.

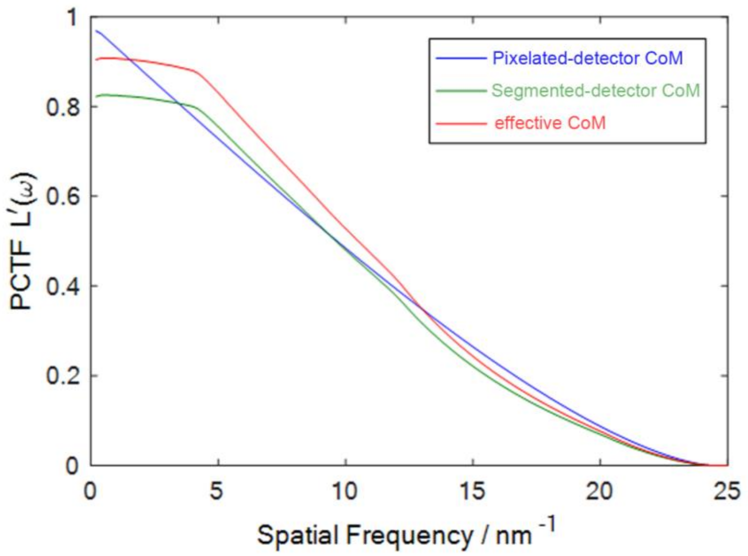

Supplementary Figure 8: PCTF using three different detector conditions.

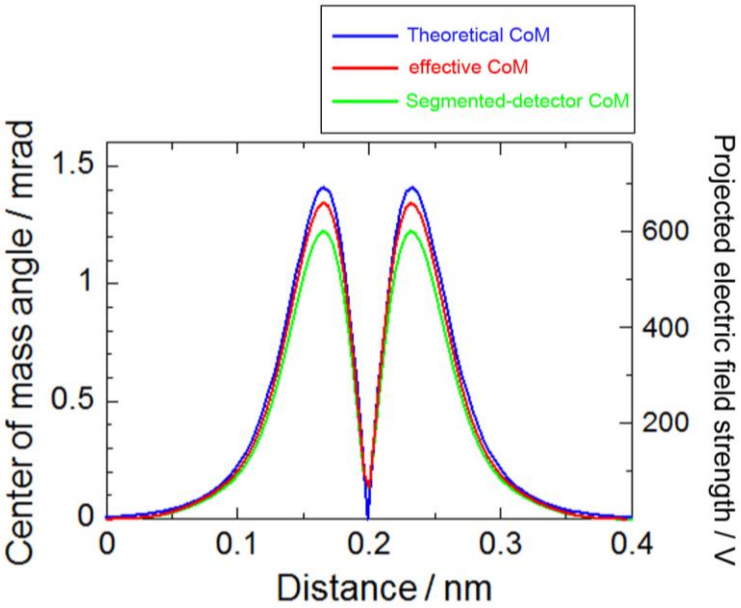

Supplementary Figure 9: Simulated electric field strength profiles of Au single atoms using different segmented detector approximations.

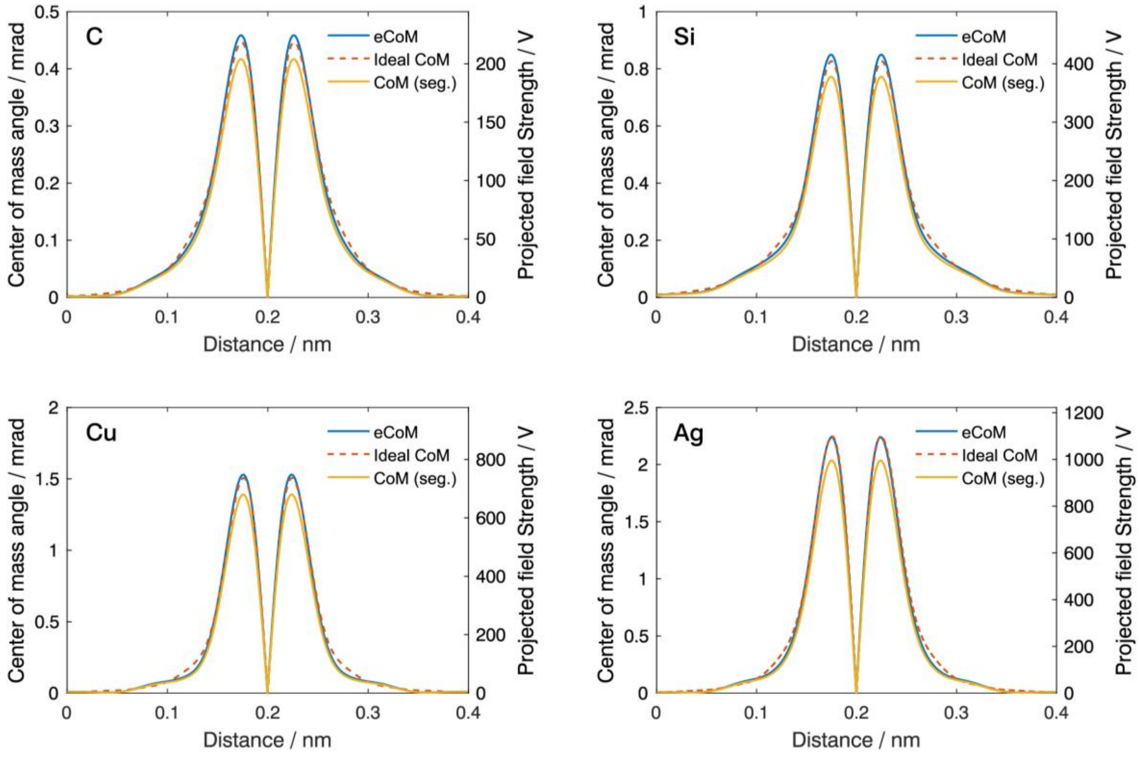

Supplementary Figure 10: Simulated electric field strength profiles of C, Si, Cu and Ag single atoms using different segmented detector approximations.

Supplementary Note 4: PACBED analysis for the sample thickness estimation for $SrTiO_3$

To experimentally estimate the sample thickness of the $SrTiO_3$ images shown in Figs. 2 and 3, we performed a position averaged convergent beam electron diffraction (PACBED) analysis. Comparing the experimental pattern with PACBED pattern simulations across a systematic range of sample thicknesses, the best match was found for a thickness of 8±1 nm. Supplementary Figure 11 shows the comparison between experimental and simulated PACBED patterns. Thus, we used the sample thickness value of 8 nm for the quantitative image simulations in the main text.

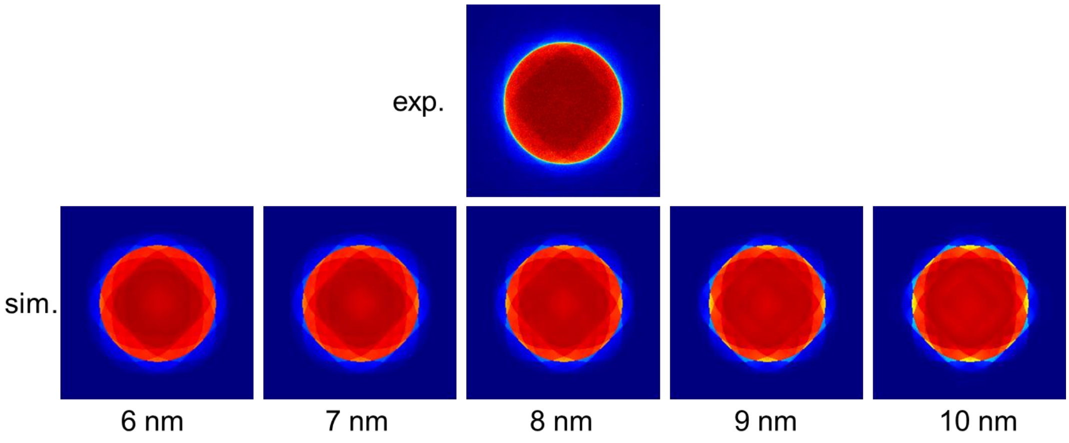

Supplementary Figure 11: Comparison between the experimental and simulated PACBED patterns used to determine the $SrTiO_3$ sample thickness for the results shown in Figs. 2 and 3. Experimental (upper panel) and simulated PACBED patterns (lower panels) of $SrTiO_3$ observed from the [001] direction. This best-match simulation has a sample thickness of 8±1 nm.

Supplementary Note 5: Finite source size and probe instability estimation for image simulations

To estimate the finite source size and probe instability effect for the DPC image simulations, we used the simultaneously obtained experimental ADF STEM images for the fitting procedure. Supplementary Figure 12 shows the experimental Sr column ADF profile and the corresponding simulated profile assuming exactly the same imaging conditions (defocus and sample thickness) used in Fig. 3 and incorporating blurring with a Gaussian effective source distribution of 0.29 Å HWHM. We see excellent agreement between experiment and simulated ADF image profile. We thus use this value of finite source size and probe instability for the $SrTiO_3$ image simulations shown in Fig. 3.

Supplementary Figure 13 shows the experimental and simulated Au single atom ADF profiles under exactly the same imaging condition used in Fig. 4. Although the experimental profile is noisier, we see good agreement between experiment and simulated ADF image profiles. Here, we assume a Gaussian effective source distribution of 0.25 Å HWHM. We use this effective source size for the Au single atom image simulations shown in Fig. 4.

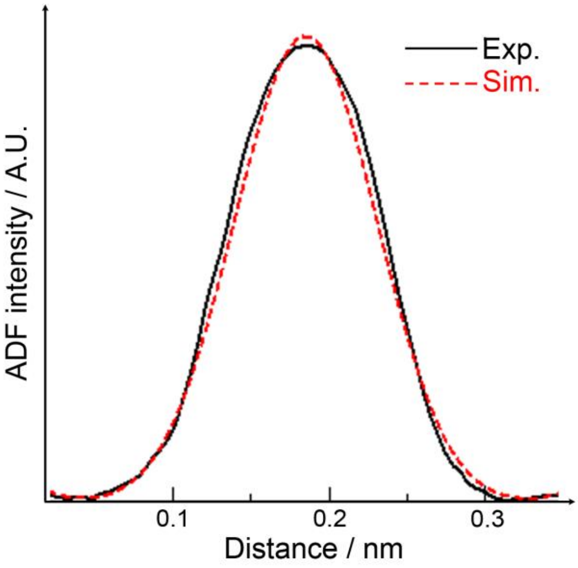

Supplementary Figure 12: Effective source size estimation for image simulations of $SrTiO_3$ shown in Fig. 3. The experimental and simulated ADF profiles of a Sr column show good agreement when we assume an effective source size of 0.29 Å HWHM in the image simulation.

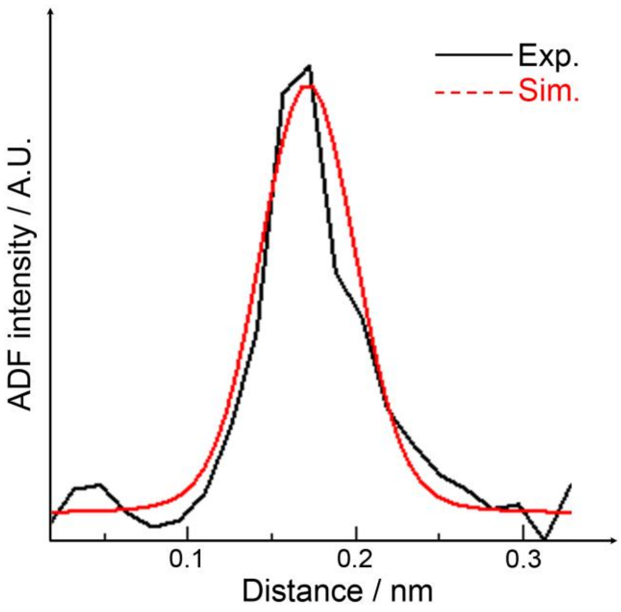

Supplementary Figure 13: Effective source size estimation for image simulations of a single Au atom shown in Fig. 4. The experimental and simulated ADF profiles of a single Au atom show good agreement when we assume an effective source size of 0.25 Å HWHM in the image simulation.

Supplementary References

1. Close, R., Chen, Z., Shibata, N. & Findlay, S.D. Towards quantitative, atomic-resolution reconstruction of the electrostatic potential via differential phase contrast using electrons. *Ultramicroscopy*, **159**, 124-137 (2015).
2. Rose, H. Nonstandard imaging methods in electron microscopy. *Ultramicroscopy*, **2**, 251-267 (1977).
