# Supplementary material for: Electric field imaging of single atoms
Source: Nat Commun. 2017 May 30;8:15631. doi: 10.1038/ncomms15631 (PMC5459997; doi:10.1038/ncomms15631)
Supplement: Peer Review File [file ncomms15631-s3.pdf]

Editorial Note: this manuscript has been previously reviewed at another journal that is not operating a transparent peer review scheme. This document only contains reviewer comments and rebuttal letters for versions considered at Nature Communications.

REVIEWERS' COMMENTS:

Reviewer #1 (Remarks to the Author):

All concerns raised in my report have been addressed in a detailed and convincing way. I suggest publication in Nature Communications.
